# Association of social capital with obesity among older adults in China: a cross-sectional analysis

**DOI:** 10.1186/s12877-022-03566-7

**Published:** 2022-11-16

**Authors:** Le Yang, Hongman Wang, Jingmin Cheng

**Affiliations:** 1 School of Management, Shanxi Medical University, 56 Xinjian South Road, Taiyuan, Shanxi Province China; 2 School of Health Humanities, Peking University, 38 Xueyuan Road, Haidian, Beijing China

**Keywords:** China, Obesity, Social capital, Older adults

## Abstract

**Background:**

Under the global aging trend, health issues of the elderly have received more and more attention. Among them, older adults’ obesity is one of the common health problems of the elderly. The association between social capital and obesity in the older adults has been analysed and discussed in previous studies but remains controversial. There are few studies on the association between social capital and obesity in the older adults in China. We examined whether social capital was associated with obesity in Chinese older adults.

**Methods:**

The data from the Chinese Longitudinal Healthy Longevity Survey (CLHLS) —wave 8 (2017–2018) was used in this study. Totally, 10,164 respondents were included in the final analysis. Generalized trust, social participation (informal social interaction and participation of organized social activities), and social support was used as measures of social capital. Obesity status was defined by body mass index (BMI). Logistic regression analyses were used to assess associations between the social capital and obesity, adjusting for confounders.

**Results:**

We found that the older adults who did not trust people around had greater odds of being overweighted/obese compared to those who trust [Adjusted Odds Ratio (AOR) 1.155, 95% CI 1.045 to 1.265]. The older adults having formal participation (participating in organized social activities) registered considerably higher BMI (AOR 1.155, 95% CI 1.041 to 1.269). The older adults who did not trust people around them had greater odds of being overweighted/obese both in males (AOR 1.172, 95% CI 1.012–1.333) and in females (AOR 1.155, 95% CI 1.004–1.306). Males having formal social participation were more likely to be overweighted/obese (AOR 1.181, 95% CI 1.024 to 1.337), but not in females.

**Conclusions:**

Generalized trust and formal social participation was associated with overweight/obesity among older adults in China. Targeted obesity interventions for older adults are needed by developing public health policies for social capital optimization.

## Background

The increase in the old population is a worldwide trend. At the end of 2019, the population of China aged 60 and above reached 253.88 million, accounting for 18.1% of the total population [1]. Due to physical and psychological vulnerability and increased risk of disease, the older adults have more complex health conditions and are currently the focus of health-related research. Among the common diseases in the older adults, obesity is particularly a common health problem [2]. Obesity is a leading metabolic risk factors contribute to increasing the risk of noncommunicable diseases (NCDs) including cardiovascular diseases such as heart disease and stroke [3–5], and has always been an important public health issue of concern to countries all over the world.

Social capital has gradually appeared in public health research as an important determinant of health. The current approaches in social capital can be roughly divided into two dimensions, social cohesion and social network. Social cohesion places more emphasis on social capital at the macro-level, such as social trust, reciprocity and norms, while the social network is mainly concerned with micro-level social capital, such as the density and scope of an individual’s social network, and different kinds of social support and resources an individual may derive from that [6, 7]. There are many different perspectives in social capital measures, including cognitive (individuals’ perceptions, values, beliefs, and attitudes) and structural (externally observable social interactions), bonding (relations between people of groups of similar social identity), bridging (relations between people of groups of different social identity) and linking (formal relations to people of power and authority) [8–11].

The association between social capital and health has always been the subject of debate and research among scholars. Due to the different measurement dimensions of social capital and the heterogeneity of research groups, there is no unified model and consensus on the measurement and research of social capital. Some studies have found that social capital has a significant positive impact on health, while other studies have found that social capital also has a negative impact on health that cannot be ignored, such as social contagion of unhealthy behavior and harmful effects (i.e. pressure) on individuals who are obsessed with social networks such that harmful social cohesion and norms are maintained [12, 13].

Many studies have been conducted to explore the association between social capital and obesity. Though Rodgers et al. (2019) [14] found that few studies proved the significant association between social capital and obesity after reviewing the literature for the period 2007–2018. Some studies reported social capital as a protective factor for overweight and obesity [15–17], while some others reported social capital as a risk factor for obesity [18, 19]. Meanwhile, many studies have also drawn different conclusions, such as a study reported that individuals with low compared to high levels of social participation were more likely to be overweight [20], but another study did not lend support to research showing a relationship between social participation and obesity [17]. The association between social capital and obesity seems to be affected by individuals, environment, social context, and cultural background. In a typical *Guanxi-based*society [21], Chinese people, especially the older generation, prioritize their family, friends and groups and appreciate traditional values, social norms and reciprocity. In addition, the different structures and arrangements of older women’s and older men’s social contexts and roles are an important source of heterogeneity that could be linked to obesity in unique ways [22]. Few studies have considered social capital and obesity and its gender differences of older adults in Chinese context. These knowledge gaps limit the effectiveness of obesity interventions for the older adults in China.

Considering the existing evidence on social capital and obesity, strong social capital could reduce the risk of obesity through promoting weight-control behaviors such as consuming low-fat diet, increasing the level of physical activity and social activity, and improving psychological well-being [16, 23–25]. However, the “dark side” of social capital also exists, such as social contagion of unhealthy behavior (smoking, drinking, and eating behaviors etc.) [13, 26, 27] and the increased unease and stress [28, 29], which increases the older adults’ obesity risk. Therefore, the purpose of this study is to explore the association between different dimensions of social capital and obesity in the older adults of China, which is vital to provide guidance to health promotion program planners and public health decision makers in obesity and chronic disease prevention in China. Using one wave of data, this study tests the following two main hypotheses: firstly, different dimensions of social capital would be associated with bodyweight status of older adults in China, secondly, there would be gender differences in the association between social capital and obesity in older adults in China.

## Methods

### Study design

The present study was a cross-sectional study to identify the relationships between social capital and obesity of the older adults in China.

### Study population

The cross-sectional data used in this paper is from publicly available source, i.e. from the Chinese Longitudinal Healthy Longevity Survey (CLHLS) —wave 8 (2017–2018) [30]. CLHLS conducted population surveys with 15,874 individuals in 2017–2018, including rural and urban areas, using a domiciliary face-to-face questionnaire, in native language. The primary target population for the survey was persons aged 65 years and above. Our analyses in this paper are based on 10,164 cases because of incomplete responses.

*BMI:* Of the 10,164 cases, body mass index (BMI) category was calculated using the standard weight status categories from WHO reference, a BMI < 18.5 kg/m^2^ is classified as underweight, a BMI of 18.5–24.9 kg/m^2^ as normal weight and a BMI of 25–29.9 kg/m^2^ as overweight, and obesity is classified as BMI ≥ 30 kg/m^2^ [31]. The average BMI was 22.48 kg/m^2^, 1598 of the older adults (15.7%) were underweight, 5980 (58.8%) had an average weight, 2197 (21.6%) were overweight, and 389 (3.8%) were obese.

*Social capital:* In public health researches on social capital and health outcomes, due to various social capital measures, the research results are also different. The results of research reports focusing on the relationship between collective social capital and health are not that clear, while social capital measures in individual-level is mostly found to be closely related to positive health outcomes [32]. The research is mainly concerned with the impact of individual-level social capital on obesity of the older adults. In this study, we combined elements of different approaches to capture individual-level social capital, such as generalized trust, social participation, and social support, which were shown as important social capital measures associated with health changes and healthcare utilization [33–35]. Social capital was captured using four variables measures: generalized trust (representing cognitive social capital); social participation, including informal social participation and formal social participation [36] (representing structural social capital); and social support (emotional and instrumental).

- 1. Question about generalized trust: Do you feel that people around are not trustworthy? The variable response is classified into 1 = untrustwforthy, 0 = trustworthy.
- 2. Question about informal social participation: Do you visit and interact with friends regularly? The variable response is divided into 1 = yes, 0 = no.
- 3. Question about formal social participation: Do you take part in social activities (organized) regularly? The variable response is divided into 1 = yes, 0 = no.
- 4. Question about social support: (1) To anyone do you talk when you need to tell something of your thoughts? (emotional support); (2) Is there anyone you could ask for help when you have problems/difficulties? (instrumental support). The variable response is grouped into 1 = yes, 0 = no.

*Covariates*: We identified potential confounders a priori from existing literature [2, 37, 38]. The first group of potential confounders were demographic and socioeconomic characteristics, including gender, age, education background, marital status, region of residence, and household annual income. Age was grouped into two categories: 65–84 and ≥ 85 years. Education background: educated and uneducated. Marital status was classified into married and living with spouse, separate, divorced, widowed, and never married. Current residential area was grouped into three categories: city, town and rural area. Household annual income was categorized as follows: low (≤ 12,000 yuan), medium (12,000–54,000 yuan), high (> 54,000 yuan).

The second group consisted of variables of health-related lifestyle, including current smoking (yes vs. no), current drinking (yes vs. no), physical activity (yes vs. no), sleep duration, staple food, and staple food intake (100 g/day). The implausible sleep duration of < 2 or > 16 h per day were regarded as unreliable and were excluded from the analysis [39]. Staple food was collapsed into five groups: rice, corn (maize), wheat (noodles, bread, etc.), rice and wheat, and other.

Lastly, we examined covariates of variables of health condition, such as depressive symptoms, activities of daily living (ADL), and cognitive function. The 10-item Center for Epidemiologic Studies Short Depression Scale (CES-D) was adopted to measure the depression symptoms, scoring from 0 to 30 [40, 41]. ADL was based on six basic daily living items, each item was scored from 1 (independent) to 3 (dependent). Cognitive function was measured using the Chinese version of Mini-Mental State Examination (MMSE), with a score ranging from 0 to 30 [42].

### Statistics

Descriptive statistics were presented as means and standard deviation (SD), or proportions. The dependent variable was set as the status of BMI (underweight, normal weight/overweight/obesity), Chi-square test was conducted to examine the significant variables. Logistic regression was performed to assess the impact of these aforementioned variables on the likelihood that the older adults would be categorized as obese, ORs and their confidence intervals were calculated for the association between each independent variable (IV) and the dependent variable. Models 1–4 examined the association between each component of social capital and BMI after controlling for different sets of confounding variables. Model 1 was generated by applying merely the social capital variables to assess the association of social capital and obesity. In Model 2, demographic and socioeconomic characteristics were controlled, and in Model 3, the study adjusted for demographic and socioeconomic characteristics and variables of health-related lifestyle. In Model 4, all the confounders were controlled. All models were tested for significance of covariates. Collinearity diagnostics were conducted in order to assess the potential for regression coefficient instability and showed a VIF range from 1.029 to 1.549, which is lower than the recommended cut-off threshold of 10. To examine gender differences in the association of social capital and obesity, we further performed stratified analyses by gender group, adjusting for all confounders.

All data were analyzed using the statistical software package IBM SPSS Statistics version 24 (IBM, Armonk, NY, USA). The level of significance was set at *P* < 0.05.

## Results

The characteristics of the study population are presented in Table 1. Approximately 47.6% of the participants were men and 52.4% were women. The proportions of female, older adults aged 65–84, educated, and married and living with spouse were relatively higher among those who overweighted or obese (*P* < 0.001). A higher percentage of older population with high household annual income was reported overweight or obese (*P* < 0.001). There were 31.7% of the older adults living in city were overweighted or obese, and that of those who living in town were 23.8% and in rural areas were 23.2%. And for those who were overweighted or obese, the proportion of rural residents were relatively higher (39.6%). There were 31.9% of the older adults who consumed wheat (noodles and bread etc.) as their staple food were overweighted or obese, and that of those who consumed corn (maize) were 31.1%, rice and wheat were 30.8%, and rice were 21.4%. Meanwhile, older adults who were overweight or obese tend to intake more staple food per day, do not smoke, drink and exercise, and get shorter sleep duration. The mean score of MMSE was relatively higher among those who overweighted or obese (*P* < 0.001). They had higher social trust, more informal social activity involved, higher social support, but less formal social activities participated.

**Table 1:** Characteristics of the older adults by BMI

	Underweight	Normal	Overweight		Obesity	*p* value
Gender, N (%)						< 0.001
Male	623(39.0%)	2973(49.7%)	1090(49.6%)		152(39.1%)	
Female	975(61.0%)	3007(50.3%)	1107(50.4%)		237(60.9%)	
Age, N (%)						< 0.001
65–84	507(31.7%)	3419(57.2%)	1632(74.3%)		272(69.9%)	
≥ 85	1091(68.3%)	2561(42.8%)	565(25.7%)		117(30.1%)	
Household annual income, N (%)						< 0.001
≤ 12,000	597(37.4%)	2046(34.2%)	689(31.4%)		131(33.7%)	
12,000–54,000	551(34.5%)	1962(32.8%)	693(31.5%)		119(30.6%)	
> 54,000	450(28.2%)	1972(33.0%)	815(37.1%)		139(35.7%)	
Education						< 0.001
Educated	593(37.1%)	2963(49.5%)	1281(58.3%)		207(53.2%)	
Uneducated	1005(62.9%)	3017(50.5%)	916(41.7%)		182(46.8%)	
Marital status, N (%)						< 0.001
Married and living with spouse	446(27.9%)	2833(47.4%)	1280(58.3%)		200(51.4%)	
Separated	38(2.4%)	113(1.9%)	38(1.7%)		7(1.8%)	
Divorced	7(0.4%)	18(0.3%)	7(0.3%)		0(0.0%)	
Widowed	1096(68.6%)	2971(49.7%)	859(39.1%)		179(46.0%)	
Never married	11(0.7%)	45(0.8%)	13(0.6%)		3(0.8%)	
Current residential area, N (%)						< 0.001
City	291(18.2%)	1375(23.0%)	649(29.5%)		124(31.9%)	
Town	571(35.7%)	1954(32.7%)	675(30.7%)		115(29.6%)	
Rural	736(46.1%)	2651(44.3%)	873(39.7%)		150(38.6%)	
Staple food, N (%)						< 0.001
Rice	1054(66.0%)	3672(61.4%)	1101(50.1%)		186(47.8%)	
Corn (maize)	59(3.7%)	211(3.5%)	97(4.4%)		25(6.4%)	
Wheat (noodles, bread, etc.)	207(13.0%)	1031(17.2%)	494(22.5%)		86(22.1%)	
Rice and wheat	259(16.2%)	1042(17.4%)	490(22.3%)		89(22.9%)	
Other	19(1.2%)	24(0.4%)	14(0.6%)		3(0.8%)	
Smoking, N (%)						< 0.001
Yes	269(16.8%)	1084(18.1%)	306(13.9%)		40(10.3%)	
No	1329(83.2%)	4896(81.9%)	1891(86.1%)		349(89.7%)	
Alcohol Drinking, N (%)						< 0.001
Yes	199(12.5%)	1003(16.8%)	377(17.2%)		48(12.3%)	
No	1399(87.5%)	4977(83.2%)	1820(82.8%)		341(87.7%)	
Physical activity, N (%)						< 0.001
Yes	379(23.7%)	2094(35.0%)	973(44.3%)		158(40.6%)	
No	1219(76.3%)	3886(65.0%)	1224(55.7%)		231(59.4%)	
Staple food intake (100 g/day), mean (SD)	2.38 (2.38)	2.71(2.53)	2.90(2.33)		2.79(3.15)	< 0.001
Sleep duration, mean (SD)	7.42(2.45)	7.36(2.14)	7.19(1.96)		7.19(2.14)	< 0.001
ADL score, mean (SD)	7.46(2.87)	6.48(1.60)	6.29(1.20)		6.51(1.60)	< 0.001
MMSE score, mean (SD)	22.14(7.69)	25.24(5.72)	26.16(4.84)		25.66(5.08)	< 0.001
Depression score, mean (SD)	7.40(3.34)	7.12(3.01)	7.21(2.96)		7.40(3.10)	< 0.01
Generalized trust, N (%)						< 0.01
Untrustworthy	234(14.6%)	845(14.1%)	379(17.3%)		70(18.0%)	
Trustworthy	1364(85.4%)	5135(85.9%)	1818(82.7%)		319(82.0%)	
Informal social participation, N (%)						< 0.001
Yes	799(50.0%)	3849(64.4%)	1503(68.4%)		241(62.0%)	
No	799(50.0%)	2131(35.6%)	694(31.6%)		148(38.0%)	
Formal participation, N (%)						< 0.001
Yes	132(8.3%)	911(15.2%)	480(21.8%)		70(18.0%)	
No	1466(91.7%)	5069(84.8%)	1717(78.2%)		319(82.0%)	
Social support (emotional), N (%)						< 0.05
Yes	1559(97.6%)	5836(97.6%)	2150(97.9%)		371(95.4%)	
No	39(2.4%)	144(2.4%)	47(2.1%)		18(4.6%)	
Social support (instrumental), N (%)						< 0.05
Yes	1586(99.2%)	5915(98.9%)	2169(98.7%)		383(98.5%)	
No	12(0.8%)	65(1.1%)	28(1.3%)		6(1.5%)	
Cases, N (%)	1598(15.7%)	5980(58.8%)	2197(21.6%)		389(3.8%)	

*P* values are calculated with analysis of Chi-squared test for categorical variables and variance (ANOVA) for continuous variables

Ordinal logistic regressions on BMI as a dependent variable were carried out (Table 2). Across all models, a significant association was found between generalized trust and social capital. The older adults who did not trust people around them had greater odds of being obese at Model 1 [Odds Ratio (OR) 1.166, 95% Confidence Interval (95% CI) 1.061 to 1.272]. After adjusting for confounders (gender, age, education background, marital status, residence area, household annual income, smoking, alcohol drinking, physical activity, sleep duration, staple food, staple food intake, depression, ADL, and cognitive function), the association were attenuated in Model 4 [Adjusted Odds Ratio (AOR) 1.155, 95% CI 1.045 to 1.265].

**Table 2:** Odds ratios (95% confidence Intervals) for respondents reporting obesity

	Model 1 (Crude OR)	Model 2^a^	Model 3^b^	Model 4^c^
Generalized trust				
Untrustworthy	1.166 ** (1.061–1.272)	1.148* (1.040–1.256)	1.149* (1.041–1.257)	1.155* (1.045–1.265)
Informal social participation				
Yes	1.406*** (1.326–1.487)	1.149*** (1.065–1.233)	1.153*** (1.066–1.239)	1.020 (0.932–1.108)
Formal social participation				
Yes	1.627*** (1.522–1.733)	1.189** (1.077–1.301)	1.170** (1.056–1.284)	1.155* (1.041–1.269)
Social support (emotional)				
Yes	1.003 (0.729–1.277)	0.896 (0.619–1.172)	0.909 (0.629–1.190)	0.920 (0.640–1.201)
Social support (instrumental)				
Yes	0.762 (0.358–1.166)	0.847 (0.439–1.255)	0.803 (0.393–1.212)	0.793 (0.383–1.203)

Results are from proportional odds models. Results are displayed as ORs of change in BMI status (contrasting increase vs constant high/low or decrease; or increase or constant high/low vs decrease) per unit increase in the original scale of generalized trust, interaction with friends, participation of organized social activities, emotional social support, or instrumental social support. ORs > 1 indicate a positive change in the outcome (i.e. overweight/obesity) as a response to an improvement of exposure

^*^p < 0.05

^**^p < 0.01

^***^p < 0.001

^a^ Adjusted for gender, age, education background, marital status, current residential area, and household annual income

^b^ Adjusted for gender, age, education background, marital status, current residential area, household annual income, smoking, alcohol drinking, physical activity, sleep duration, staple food, and staple food intake

^c^ Adjusted for gender, age, education background, marital status, current residential area, household annual income, smoking, alcohol drinking, physical activity, sleep duration, staple food, staple food intake, depression, ADL, and cognitive function

Among the informal social participation (interaction with friends) categories, the older adults interacting with friends (OR 1.406, 95% CI 1.326 to 1.487) registered considerably higher BMI compared to those who did not at Model 1, and slightly lower odds were observed in Model 2 and 3, and this association was further mediated when variables of health condition (Model 4) were added.

And significant association between formal social participation (participation of organized social activities) and obesity was found, the group who participated organized social activities had 62.7% higher odds of being obese in Model 1 (OR 1.627, 95% CI 1.522 to 1.733), and the odds was decreased slightly after controlling for all potential confounders in Model 4 (AOR 1.155, 95% CI 1.041 to 1.269).

We further examined the association between social capital and bodyweight status by gender. As shown in Table 3, formal social participation significantly increased the odds of being obese but to a trivial extent for males (AOR 1.181, 95% CI 1.024 to 1.337), but not females. The older adults who did not trust people around them had greater odds of being obese both in males (AOR 1.172, 95% CI 1.012–1.333) and females (AOR 1.155, 95% CI 1.004–1.306).

**Table 3:** Odds ratios (95% confidence Intervals) for respondents reporting obesity by gender

	male	female
Generalized trust		
Untrustworthy	1.172* (1.012–1.333)	1.155* (1.004–1.306)
Interaction with friends		
Yes	1.069 (0.940–1.199)	0.971 (0.850–1.093)
Participation of organized social activities		
Yes	1.181* (1.024–1.337)	1.157 (0.991–1.324)
Social support (emotional)		
Yes	0.696 (0.244–1.149)	1.104 (0.747–1.461)
Social support (instrumental)		
Yes	0.824 (0.221–1.428)	0.779 (0.216–1.341)

Results are from proportional odds models. Results are displayed as ORs of change in BMI status (contrasting increase vs constant high/low or decrease; or increase or constant high/low vs decrease) per unit increase in the original scale of generalized trust, interaction with friends, participation of organized social activities, emotional social support, or instrumental social support. ORs > 1 indicate a positive change in the outcome (i.e. overweight/obesity) as a response to an improvement of exposure

Adjusted for age, education background, marital status, current residential area, household annual income, smoking, alcohol drinking, physical activity, sleep duration, staple food, staple food intake, depression, ADL, and cognitive function

^*^ *p* < 0.05

## Discussion

Our findings suggest that generalized trust and formal social participation have a significant relationship with obesity among older adults in China, but does not lend support to research showing a relationship between social support and obesity [43, 44].

*Generalized trust*: As far as we know, the finding reported in the present study is the first to investigate associations between generalized trust and the older adults’ obesity in China. We find evidence that low level of generalized trust was significantly associated with higher odds of obesity. The finding is somewhat consistent with the findings of Wu et al. [45], in their study, generalized trust was associated with lower risk of obesity. There could be at least two interpretations of the association between generalized trust and the older adults’ obesity. The first is social capital’s buffering of psychological pressure [46, 47]. When the generalized trust level of the older adults is low, they are more likely to feel uneasy and lonely, their psychological pressure cannot be effectively buffered by social capital, so there is a relatively high possibility of obesity caused by stress-related eating [48]. Second, a low level of generalized trust leads to a lower level of safety among residents and poor social control [49]. Several studies had found the feeling of insecurity was associated with overweight/obesity, for people living in unsafe environment had shorter physical activity duration, which might cause a gain in BMI [50–52].

*Social participation*: What the research found is the negative effect of formal social participation on obesity. The finding indicates that participating in organized social activities increases the likelihood of being obese in the older adults of China. Many studies have confirmed the social contagion of unhealthy behaviors in social interactions [53–56]. From the perspective of social contagion, unhealthy eating behavior, such as unhealthy dietary habits, can be promoted among the older adults through frequent social interaction, especially in the context of Chinese social culture, people always socialize through meals, which increases the risk of being obese. In the other hand, from the perspective of “obesity paradox”, a high body mass index (BMI) is associated with lower mortality and could be protective for older adults’ health [36, 57]. Hence, the older adults who are overweight may had better health conditions and more likely to be involved in social activities. Furthermore, the obesity measurement also takes weight, this study used BMI alone to measure the obesity in the older adults, the older adults with good physical condition and large amounts of muscle mass and were more active in social participation may be classified as overweight [58].

It should also be noted that the differences in the association between social capital and obesity as to different genders under the influence of factors such as traditional social roles and social responsibilities in China. Women have more close relationship with their family members and friends, while men tend to rely more on social connections through the workplace and participation in organizations [59]. Hence, the reasons and mechanism for relationship between social participation and obesity in different genders may be different and needs to be explored in future studies.

Several limitations should be noted in this study. Our findings are based on cross-sectional data; thus, no causal relationships could be established. We are not able to measure social capital comprehensively due to lack of data. Without such information, the accuracy of our estimation on the association between social capital and obesity would be compromised. In addition, the obesity measurement in this study is limited. Last, although we discovered that formal social participation played an important role in obesity for males and not for female, the underlying mechanisms were still speculative and future research can go deep along this line of thought. Despite these limitations, our findings contribute to the limited body of existing literature regarding social capital and overweight/obesity. We found that generalized trust and formal social participation was associated with overweight/obesity, which could provide some evidence base for obesity interventions in the older adults.

## Conclusions

As the prevalence of overweight and obesity are increasing in older adults, our study explores the association between social capital and obesity in Chinese older adults. Given the results of our study, low level of generalized trust and participation of organized social activities were associated with higher BMI, even adjusting for their demographic and socioeconomic status, health-related lifestyle, and health conditions. In addition, the formal social participation was significantly associated with higher BMI for the males. Targeted obesity interventions are needed by considering public health policies aiming at social trust, and more longitudinal studies are in need to examine the reliability and validity of the outcomes and the effect mechanism of social capital on obesity in older adults.

## Data Availability

PKU Centre for Healthy Ageing and Development. 2021. “Chinese Longitudinal Healthy Longevity Survey (CLHLS).” Peking University Open Research Data. https://doi.org/10.18170/DVN/WBO7LK.

## Abbreviations

CLHLS: Chinese Longitudinal Healthy Longevity Survey
BMI: Body Mass Index
AOR: Adjusted Odds Ratio
NCDs: Noncommunicable Diseases
SD: Standard Deviation
OR: Odds Ratio
95% CI: 95% Confidence Interval
